# Transcriptional Profile of Soybean Seeds with Contrasting Seed Coat Color

**DOI:** 10.3390/plants12071555

**Published:** 2023-04-04

**Authors:** João M. Kafer, Mayla D. C. Molinari, Fernando A. Henning, Alessandra Koltun, Viviani V. Marques, Silvana R. R. Marin, Alexandre L. Nepomuceno, Liliane M. Mertz-Henning

**Affiliations:** 1 Biotechnology Department, Londrina State University, Londrina 86057-970, PR, Brazil; 2 Arthur Bernardes Foundation, Embrapa Soja, Londrina 86085-981, PR, Brazil; maylamolinari@hotmail.com (M.D.C.M.); vivianimarques@yahoo.com.br (V.V.M.); 3 Embrapa Soja, Londrina 86085-981, PR, Brazil; fernando.henning@embrapa.br (F.A.H.); silvana.marin@embrapa.br (S.R.R.M.); alexandre.nepomuceno@embrapa.br (A.L.N.); 4 Agronomy Department, State University of Maringá, Maringá 87020-900, PR, Brazil; koltun@alumni.usp.br

**Keywords:** *Glycine max*, soybean storage, RNA-Seq, lignin

## Abstract

Soybean is the primary source of vegetable protein and is used for various purposes, mainly to feed animals. This crop can have diverse seed coat colors, varying from yellow, black, brown, and green to bicolor. Black seed coat cultivars have already been assigned as favorable for both seed and grain production. Thus, this work aimed to identify genes associated with soybean seed quality by comparing the transcriptomes of soybean seeds with contrasting seed coat colors. The results from RNA-seq analyses were validated with real-time PCR using the cultivar BRS 715A (black seed coat) and the cultivars BRS 413 RR and DM 6563 IPRO (yellow seed coat). We found 318 genes differentially expressed in all cultivars (freshly harvested seeds and seeds stored in cold chamber). From the in silico analysis of the transcriptomes, the following genes were selected and validated with RT-qPCR: *ACS1*, *ACSF3*, *CYP90A1*, *CYP710A1*, *HCT*, *CBL*, and *SAHH*. These genes are genes induced in the black seed coat cultivar and are part of pathways responsible for ethylene, lipid, brassinosteroid, lignin, and sulfur amino acid biosynthesis. The BRSMG 715A gene has almost 4times more lignin than the yellow seed coat cultivars. These attributes are related to the BRSMG 715A cultivar’s higher seed quality, which translates to more longevity and resistance to moisture and mechanical damage. Future silencing studies may evaluate the knockout of these genes to better understand the biology of soybean seeds with black seed coat.

## 1. Introduction

Soybean (*Glycine max* (L.) Merrill) is one of the main agricultural commodities worldwide, possessing great economic importance and showing ever-increasing production. Its wide popularity comes largely from the fact that its grains have one of the highest protein contents (approximately 40%) and third largest oil content (approximately 20%) [1,2,3]. Currently, Brazil, the United States, Argentina, China, and India are the top producing countries [4,5].

This valuable leguminous species is used for various purposes, such as in animal feed, human food, in the chemical industry to manufacture cosmetics and fibers, and in the production of biodiesel, among other uses. Therefore, soybean production occurs all over the world with a planted area of 127 million hectares worldwide and a forecasted global production of 350 million tons for 2022 [5].

One of the main factors that contributed to the success of soybean cultivation was the development of high-yield cultivars adapted to different regions. Despite the large number of soybean cultivars available, there is low genetic variability among genotypes, mainly due to the narrow genetic base of the germplasm [6]. Nevertheless, it is possible to find variability in the species regarding several characteristics, including seed coat color, which can vary from yellow, green, black, and brown to bicolor [7]. In addition, coat color can indicate other traits with potential added value to commercial products, either to identify genes of interest to be incorporated into genetic improvement programs or to be used as raw materials for new products or technologies.

Although most commercial soybean cultivars have a yellow seed coat, the black seed coat of soybeans has properties that make it attractive both for seed and grain use. For instance, higher concentrations of phenolic compounds such as anthocyanins, carotenoids, and lignin are found in black-seeded compared to yellow-seeded soybeans [8,9].

Thus, concerning grains, the black seed coat soybean is an attractive alternative for human consumption, representing a great source of several essential nutrients [1]. In addition, it presents a wide variety of phytochemical substances—highlighting isoflavonoids, phenolic acids, saponins, and triterpenoids—that benefit human health [10]. Furthermore, soybean seeds with black seed coats may have high levels of anthocyanins (antioxidants), which may have many health benefits, including preventing diseases such as cancer [11,12]. In addition to acting as antioxidants, anthocyanins have anti-inflammatory, nephroprotective, antidiabetic, anti-infertility, anti-obesity, anti-arthritic, neuroprotective, anti-hyperlipidemic, anti-cataract, and healing properties [13].

Regarding seed quality, it has already been observed that genotypes with high levels of lignin in their seed coats present greater thickness and, consequently, greater tissue resistance to possible physical damage due to moisture, mechanical damage, and/or the presence of pathogens [14,15,16,17,18,19]. Lignin is also associated with more thickness in other tissues. In pods, higher lignin content is associated with less weathering damage and, consequently, less damage in seeds during maturation and harvesting [20]. In roots and leaves, lignin plays a role in adaptation against drought, salinity, and temperature variation [21,22]. Another relevant factor is that soybean seeds with black seed coats generally have better physiological quality than those with yellow seed coats. Finally, black-seeded soybeans are associated with better longevity during storage [17,23,24,25].

Soybean seed coat pigmentation is a complex trait mainly governed by five interacting loci (*I*, *R*, *T*, *W1*, and *O*) [7]. Nevertheless, studies have shown that green-coated seeds involve different segregation patterns compared to other loci, such as *G1*, *G2*, and *G3* [23]. Mechanisms of post-transcriptional gene silencing mediated by small interfering RNA (siRNA) also control this trait [26].

Many other beneficial molecules may be present and associated with black-seeded soybean cultivars; however, they need to be explored on a large scale to identify promising genes for crop improvement or even to develop new ones, aiming at products with added value.

Given the above, this study of the transcriptomes of soybean seeds with seed coats of contrasting colors will advance our knowledge of the genetic–molecular mechanisms that confer superior characteristics to black seeds and grains.

## 2. Results and Discussion

### 2.1. Differential Expression

**Figure 1:**
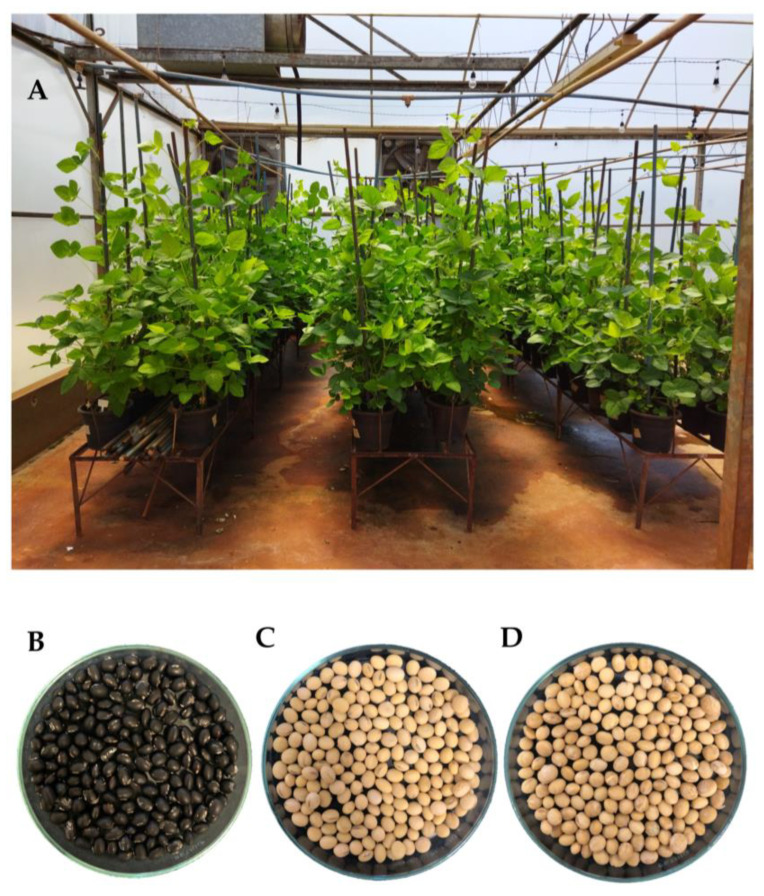
Experiment conducted in greenhouse to collect material for RNA extraction (**A**). Seeds of the BRSMG 715A genotype (**B**). Seeds of the BRS 413 RR genotype (**C**). Seeds of the DM 6563 IPRO genotype (**D**).

To explore the differences between the transcriptomes of seeds with contrasting tegument coloration, three cultivars were selected, the cultivar BRSMG 715A (Figure 1B) representing genotypes of black seed coat coloration and the cultivars BRS 413 RR (Figure 1C) and DM 6563 IPRO (Figure 1D) representing yellow seed coat color genotypes. Two identical experiments were conducted to collect material for RNA extraction for RNA sequencing (RNA-Seq) and Reverse Transcription quantitative Polymerase Chain Reaction (RT-qPCR) in a greenhouse and were used to compose two treatments (Figure 1A): freshly harvested seeds (FHS) and seeds stored in a cold chamber for six months (SCC) (for more details, see the Section 3).

**Figure 2:**
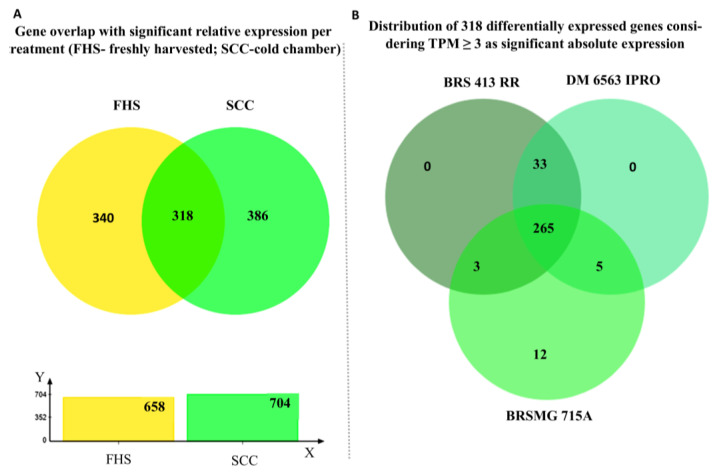
Venn diagrams considering genes with significant relative (**A**) and absolute (**B**) expression in soybean. In A, an overlap of genes with a significant relative expression between the black seed coat cultivar BRSMG 715A and the yellow cultivars BRS 413 RR and DM 6563 iPRO per treatment (FHS—freshly harvested and SCC—cold room). In (**B**), the overlap among cultivar BRSMG 715A and cultivars BRS 413 RR and DM 6563 iPRO for the 318 genes with significant absolute expression (TPM ≥ 3) common to FHS and SCC is shown. Only genes with significant relative expression (log2FC ≤ −1 and ≥ 1) common to FHS and SCC (318) in (**A**) were evaluated for their absolute expression (**B**).

In the treatment of freshly harvested seeds (FHS), 658 genes were identified as differentially expressed in the black seed coats of the cultivar BRSMG 715A compared to the yellow seed coat cultivars (BRS 413 RR and DM 6563 IPRO). Additionally, 340 of these genes were expressed exclusively in this treatment. In the second treatment, seeds were stored in a cold chamber for six months (SCC), and 704 differentially expressed genes were identified; from this total, 386 were expressed exclusively in this treatment (Figure 2A). The two treatments shared 318 genes (Figure 2A; Supplementary Material File S3). Therefore, to identify genes that could be involved only in the differences between cultivars, the gene cluster of 318 genes was used for gene selection via RT-qPCR (Figure 2A).

The differentially expressed genes were divided into two subgroups based on their absolute expression values in transcripts per million (TPM). The first group refers to genes with significant absolute expression (TPM ≥ 3), and the second group refers to genes with non-significant or absent absolute expression (TPM < 3) (Supplemental Material File S2). Only genes with significant absolute expression in all genotypes were used for RT-qPCR validation.

Figure 2B shows a Venn diagram subdividing the group of genes with significant absolute expression, wherein we found 265 common genes for all cultivars, 33 genes in yellow cultivars, 3 common genes between the yellow cultivar BRS 413 RR and the black cultivar, 5 common genes between the yellow cultivar DM 6563 IPRO and the black cultivar, and 12 genes exclusive to the black seed coat cultivar (BRSMG 715A).

The exclusively expressed genes of the black seed coat cultivar are shown in Table 1. Glyma.06G213600 encodes an isoamylase enzyme that performs starch debranching, transforming starch into maltodextrin [27,28]. Glyma.06G043900 encodes cyclin A, an important protein for embryo differentiation [29]. The Glyma.01G016700 gene is an inositol-pentakisphosphate 2-kinase that is associated with phytic acid (considered an antinutritional mineral-chelating factor) and is one of the candidates for reducing the substance through knockout and silencing. However, this gene is also necessary for seedling growth and phosphate homeostasis [30,31]. The Glyma.02G064300 gene encodes the t2 ribonuclease enzyme and is involved in nodulation processes in soybeans [32]. Further, four genes are related to disease resistance; among these, three are leucine-rich repeat receptors (LRRs) and are involved in recognizing pathogen signals, plant phytoregulators, and developmental signals [33]. The Glyma.20G138000 gene encodes a cysteine-rich repeat associated with disease resistance [34]. Finally, a phosphatidylinositol glycan class s (PIGS) is encoded by Glyma.20G138000. It participates in protein glycolysis, an important action for several biological processes, such as the quality control of proteins in the endoplasmic reticulum, stability, and interactions between proteins [35]. These genes can be used in further studies for overexpression and/or silencing to validate their functions in seed quality.

The relative expression of the differentially expressed genes mentioned in Figure 2A is described in Supplementary Material File S2. Out of a total of 1044 genes, 658 expressed in the FHS treatment and 704 expressed in the SCC treatment (Figure 2A). Of the 658 genes expressed in FHS, 341 were down-regulated and 317 were up-regulated in the black cultivar. Of the 704 genes expressed in SCC, 351 were down-regulated, and 353 were up-regulated in the black cultivar (Supplementary Material File S2). All 1044 genes evaluated are present in the two versions of the Williams 82 reference genome (Wm82.a2; Wm82.a4).

### 2.2. Enriched Metabolic Pathways

**Figure 3:**
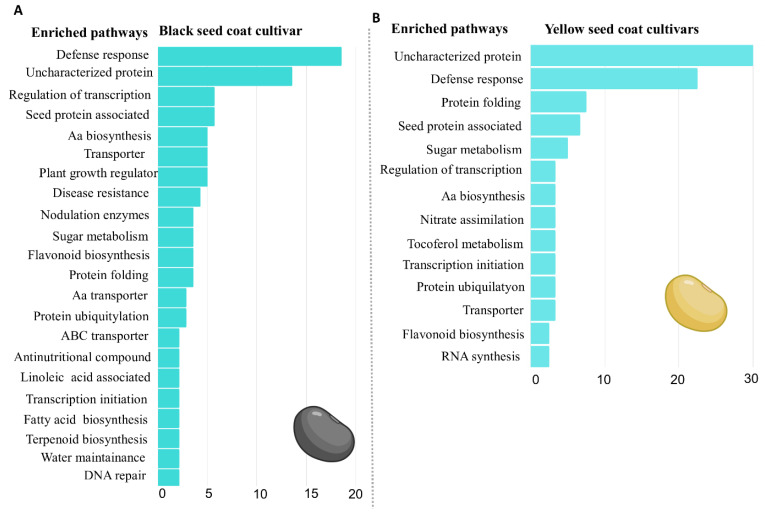
Metabolic pathways enriched in cultivar BRSMG 715A of soybeans in FHS and SCC (**A**) and in cultivars BRS 413 RR and DM 6563 IPRO in FHS and SCC (**B**).

The group of genes with significant absolute expression (TPM ≥ 3) was used to identify the metabolic pathways enriched in black and yellow seed coat cultivars. Only pathways enriched with at least three genes were considered (Figure 3).

The black seed coat cultivar BRSMG 715A presented a total of 22 enriched routes in its seeds, while the yellow seed coat cultivars presented only 14 routes (Figure 3). For better organization, the 22 enriched pathways were separated into two gene groups: (Group A) number of genes above 4% and (Group B) below 4%.

In group A, 18.57% of genes are involved in defense responses, 13.57% are uncharacterized proteins, 5.71% participate in transcriptional regulation, 5.71% are associated with the protein content in the seed, 5% are related to amino acid biosynthesis, 5% are transporters, 5% are associated with plant growth regulator biosynthesis, and 4.29% are genes responsible for disease resistance. In group B, 3.57% regulate enzymes responsible for root nodulation, 3.57% are involved in sugar metabolism, 3.57% participate in flavonoid biosynthesis, 3.57% are responsible for protein folding, 2.86% are amino acid transporters, 2.86% take part in the protein ubiquitylation process, 2.14% belong to the ABC conveyor class, 2.14% are antinutritional compounds, 2.14% are related to linolenic acid metabolism, 2.14% are transcription initiators, 2.14% participate in fatty acid biosynthesis, 2.14% are related to terpenoid biosynthesis, 2.14% belong to water-maintaining proteins in the cell, and 2.14% are involved in DNA repair (Figure 3A).

Among the enriched pathways common in the yellow cultivars BRS 413 RR and DM 6563 IPRO (Figure 3B), 30% are uncharacterized proteins, 22.50% are associated with defense responses, 7.5% are involved in the protein folding process, 6.67% are associated with seed protein content, and 5% participate in sugar metabolism. Nevertheless, 3.33% are related to transcriptional regulation, 3.33% are associated with amino acid biosynthesis, 3.33% participate in nitrate assimilation, 3.33% are associated with tocopherol biosynthesis, 3.33% are transcription initiators, 3.33% take part in protein ubiquitylation, 3.33% participate in the transport of solutes in cells, 2.5% are associated with flavonoid biosynthesis, and 2.5% are related to RNA biosynthesis (Figure 3B).

The results obtained under these conditions led to the identification of 11 enriched pathways exclusive to the black seed coat cultivar BRSMG 715A, namely, plant growth regulator biosynthesis, disease resistance, nodulation enzymes, amino acid transport, ABC transporters, antinutritional compounds, linoleic acid associated, fatty acid biosynthesis, terpenoid biosynthesis, maintenance of water content, and DNA repair. These pathways represent approximately 30.7% of the enriched pathways. In the yellow cultivars (BRS 413 RR and DM 6563 IPRO), only three exclusive pathways were identified: nitrate assimilation, tocopherol metabolism, and RNA synthesis. In the cultivars with yellow seed coats, the enriched routes represented approximately 9.16%.

In addition, 11 enriched pathways were common for the three cultivars evaluated, showing differences between the number of genes found. The black seed coat cultivar BRSMG 715A has 2.38% more genes associated with transcriptional regulation, 1.67% more genes related to amino acid biosynthesis, 1.67% more transporters, and 1.07% more genes associated with flavonoid biosynthesis. The cultivars DM 6563 IPRO and BRS 413 RR with yellow seed coats showed 16.43% more genes encoding uncharacterized proteins, 3.93% more genes associated with defense response, 3.93% more genes related to protein folding, 1.43% more genes that participate in sugar metabolism, 1.19% that are transcription initiators, 0.96% more genes related to seed protein content, and 0.47% more genes involved with protein ubiquitination.

### 2.3. Relative and Absolute Expression

**Figure 4:**
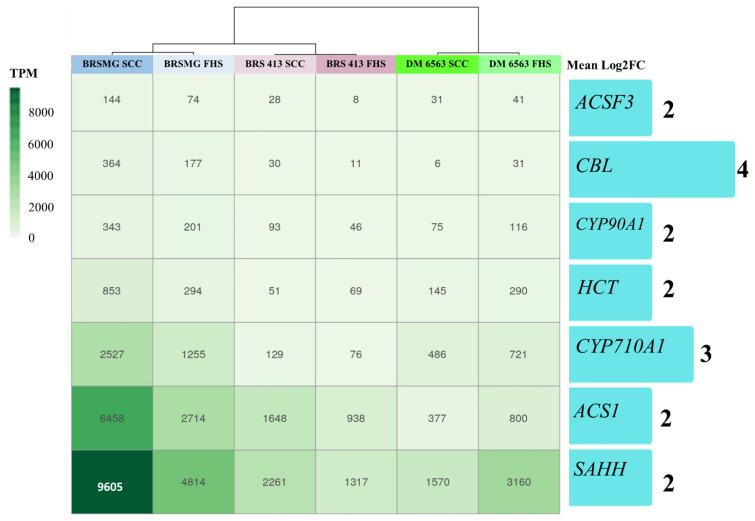
Heatmap containing absolute expression values in transcripts per million (TPM). Bars represent the relative expression in log2FC of the seven target genes. The selected genes were all up-regulated in the black seed coat cultivar BRSMG 715A of soybeans. The green boxes show the TPM values used to calculate the log2FC for each gene (blue bar).

RNA-seq data were validated via Reverse Transcription—quantitative Polymerase Chain Reaction (RT-qPCR). Seven genes with expression in TPM ≥ 3 were selected: genes involved in (a) amino acid biosynthesis (*CBL* and *SAHH*), (b) plant growth regulator biosynthesis (*ACS1*, *CYP90A1*, and *CYP710A1*), (c) fatty acid biosynthesis (*ACSF3*), and (d) lignin synthesis (*HCT*). These genes were common in treatments FHS and SCC and for the three cultivars. The identified genes showed interesting biological functions that were associated with the characteristics attributed to black cultivars, such as higher physiological seed quality, higher protein quality, and health-beneficial compounds [16,36,37]. Considering the absolute expression set (TPM) of the seven genes analyzed, greater similarity was observed between treatments (FHS and SCC) within each cultivar (Figure 4). As a result, three clusters (blue, purple, and green) were generated. These data show that the expression of target genes is similar in both treatments.

Regardless of the variation in absolute expression (TPM), all genes were, on average, 4 times more expressed (4×) (log2FC = 2) in the black seed coat cultivar than in the yellow ones.

These 7 genes were divided into four groups according to their biological function: genes involved in (a) amino acid biosynthesis (*CBL* and *SAHH*), (b) plant growth regulator biosynthesis (*ACS1*, *CYP90A1*, and *CYP710A1*), (c) fatty acid biosynthesis (*ACSF3*), and (d) lignin synthesis (*HCT*).

#### 2.3.1. Genes Involved in Amino Acid Biosynthesis

The *CBL* and *SAHH* genes that are involved in the sulfur amino acids methionine and cysteine biosynthesis were selected. Methionine and cysteine are considered essential amino acids, as they are not produced in the human body, and therefore must be acquired through one’s diet. Its consumption is essential for the biosynthesis of many molecules important for the correct performance of various organisms, such as creatine, carnitine, polyamines, epinephrine, choline, and melatonin [38,39]. Furthermore, in the biosynthesis of sulfur amino acids, the product, S-adenosylmethionine, is a precursor for the methylation of monolignols, an essential process for lignin formation [40].

**Figure 5:**
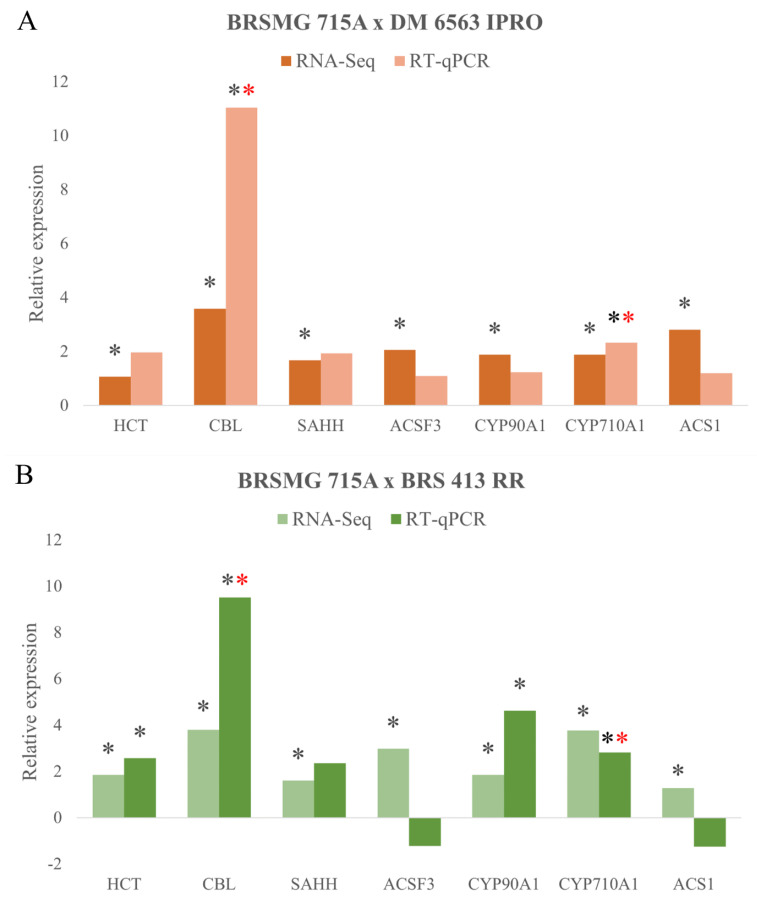
Relative expression of the *HCT*, *CBL*, *SAHH*, *ACSF3*, *CYP90A1*, *CYP710A1*, and *ACS1* genes in freshly harvested whole soybean seeds. Positive values represent expression in the black seed coat cultivar BRSMG 715A compared to the yellow seed coat cultivars BRS 413 RR (**A**) and DM 6563 IPRO (**B**). For RT-qPCR (2^−ΔΔCt^), the statistical analysis was performed using the *t*-test (*p* ≤ 0.1) and Wilcox test (*p* ≤ 0.05). For RNA-Seq (log2FC) analysis, FDR ≤ 0.05 was used. Asterisks (*) represent values with a significant difference. Red asterisks represent significant values according to the Wilcox test.

The *CBL* gene, which was expressed 16× more in the black seed coat cultivar (Log2FC = 4) according to RNA-Seq analysis, showed the same pattern of regulation via RT-qPCR analysis (Figure 5), with 11 and 9×x more expression in the black seed coat cultivar BRSMG 715A than in the yellow seed coat cultivars DM 6563 IPRO and BRS 413 RR, respectively. The gene encodes the enzyme cystathionine beta-lyase, which performs interconversions between L-homocysteine and L-cysteine. The *CBL* gene is a promising candidate for increasing cysteine and methionine in soybeans [36,41]. It has been shown, by means of RNAi, that gene repression causes a reduction in methionine biosynthesis, which results in lower growth and development in potatoes [42]. Furthermore, through gene silencing, it was verified that this gene is crucial for embryo and root development in *Arabidopsis*, affecting the regulation of gene expression and methionine biosynthesis [43].

The *SAHH* gene encodes S-adenosil homocysteinase hydrolase. This enzyme transforms S-adenosyl-L-homocysteine into L-homocysteine and adenosine, which is important for cell growth and regulation of gene expression through DNA methylation [44]. *SAHH* was 4× more expressed in the black seed coat cultivar BRSMG 715A (Log2FC = 2) than in the yellow seed coat cultivars. In the relative expression observed via RT-qPCR analysis, this gene showed no significant difference between the black seed coat cultivar and the yellow-coated seed cultivars. However, they followed the same expression pattern that was observed via RNA-Seq analysis. Additionally, S-adenosil homocysteinase hydrolase is involved in lignin formation since it transforms the substrate S-adenosyl homocysteine (an inhibitor of monolignol methylation) into L-homocysteine; thus, its expression is essential for lignin biosynthesis [40]. This enzyme is also involved in methionine recovery through the formation of the L-homocysteine product via de novo methionine synthesis [45]. Deficiency in one or more amino acids is sufficient to negatively impact the growth and development of different organisms [38]. Therefore, one of the focuses of genetic improvement has been to enrich the composition of the amino acids present in soybeans and not just the crude protein content itself.

#### 2.3.2. Genes Involved in Plant Growth Regulators

Regarding plant growth regulators, genes involved in ethylene and brassinosteroid biosynthesis were identified. The *ACS1* gene encodes the 1-aminocyclopropane-1-carboxylate synthase, one of the main enzymes responsible for ethylene biosynthesis. This gene showed 4× higher expression in the black seed coat cultivar BRSMG 715A (log2FC = 2) via RNA-Seq analysis. In the analysis of relative expression via RT-qPCR, *ACS1* expression showed no significant difference between the black seed coat cultivar BRSMG 715A and the yellow seed coat cultivars DM 6563 IPRO and BRS 413 RR (Figure 5AB). However, *ACS1* showed higher expression in the cultivar BRS 413 RR (Figure 5B). Ethylene is one of the plant hormones that influences plant growth and development and biotic and abiotic stress responses. *ACS1* works by converting the substrate D-adenosyl-methionine into 1-aminocyclopropane-1-carboxylic acid (*ACC*), which is subsequently transformed into ethylene by the enzyme aminocyclopropane-1-carboxylate oxidase (*ACO*) [46]. Ethylene is one of the hormones responsible for germination, increasing its concentration and inducing radicle growth to a borderline point, subsequently reducing seedling growth [47]. Furthermore, ethylene participates in the formation, maintenance, and elongation of the plumular hook. It also participates in hypocotyl elongation [48].

The *CPD* gene encodes a c-3 oxidase (*CYP90A1*) that acts in the biosynthesis of brassinosteroids (BRs), catalyzing the reaction between (22S)-22-hydroxycampesterol and (22R,23R)-22,23-dihydroxycampesterol, as well as the reaction between the substrate 6-deoxocatasterone and the product 6-desoxoteasterone. The *CYP90A1* gene was 4× more expressed in the black seed coat cultivar than in the yellow cultivars (log2FC = 2). In the analysis of relative expression via RT-qPCR, *CYP90A1* showed a significant difference between BRSMG 715A (black seed coat) and BRS 413 RR (yellow seed coat). However, no significant difference was observed for *CYP90A1* relative expression between BRSMG 715A and DM6563 IPRO. Nevertheless, the *CYP90A1* regulation found in RNA-Seq was maintained (Figure 5A,B).

In *cpd* mutants, the synthesis of BRs is interrupted in the step prior to their activity [49]. In the seed, BRs promote germination and act in contrast to abscisic acid, which inhibits the process. In soybeans, the most active form of BR promotes epicotyl and hypocotyl elongation. Furthermore, BRs delay leaf senescence and induce genes that modify cell walls and AIA-related genes while suppressing *WRKY* transcription factors involved in senescence and stresses [50]. *CPD* is essential for plant development; the absence of this gene causes dwarfism, and *CPD* complementation recovers normal plant development [51]. Furthermore, it was found that the action of BRs can increase lignin as well as alter the expression of genes involved in phenylpropanoid biosynthesis [52,53].

The *CYP710A1* gene encodes a sterol 22-desaturase, one of the main enzymes in plant sterol biosynthesis and a brassinosteroid precursor. It acts at the end of the plant sterol pathway, giving rise to brassicasterol and stigmasterol products. *CYP710A1* showed 6× higher relative expression in the black seed coat cultivar BRSMG 715A (log2FC = 3). In the analysis of relative expression via RT-qPCR in BRSMG 715A, this gene showed a significant difference compared to BRS 413 RR but did not differ in relation to DM6563 IPRO. Finally, the *CYP710A1* regulation pattern observed in the RNA-Seq was maintained (Figure 4A,B). This enzyme introduces a double bond at C22 of β-sitosterol, transforming it into stigmasterol [54,55]. Interconversions between β-sitosterol and stigmasterol are associated with tolerance to biotic and abiotic stresses by modifying the permeability of plant cell membranes [56,57,58]. In cottonseed, this gene is induced during plant development together with other genes that participate in the plant sterol pathway [59]. Moreover, stigmasterol has several benefits for human health, and the overexpression *CYP710A1* generated a 2.6× increase in this compound in tomato fruit [37]. In addition, this gene was identified in QTLs as a candidate for increasing the number of pods per plant, as well as other genes of the BR pathway that influence the growth and development of soybean pods [60,61].

#### 2.3.3. Genes Involved in Fatty Acid Biosynthesis

Regarding *ACSF3*, the gene encodes the malonyl-CoA/methylmalonyl-CoA synthetase enzyme that catalyzes the reaction between the malonate substrate and the malonyl-CoA product. This product is one of the main precursors for synthesizing and elongating fatty acids. Additionally, it is necessary for the formation of other compounds, such as phytoalexins, flavonoids, and anthocyanins. *ACSF3* was 4× more expressed in the black seed coat cultivar BRSMG 715A (log2FC = 2). In the relative expression observed in the RT-qPCR analysis, this gene showed no significant difference compared to the yellow seed coat cultivars DM6563 IPRO and BRS 413 RR. However, it showed higher relative expression in BRS 413 RR (Figure 5B). *ACSF3* is considered essential for plant development and growth; in *Arabidopsis*, mutants deficient in this enzyme showed retarded growth and accumulated malonic and succinic acid [62]. Malonyl-CoA is one of the necessary precursors for the *CHS* enzyme (the first enzyme in the flavonoid and isoflavonoid formation pathway), which condenses three molecules of malonyl-CoA together with a molecule of p-coumaroyl-CoA. This reaction gives rise to the other flavonoids present in soybean seeds [63]. Among them, the products from the flavonoid pathway are anthocyanins, which result in black pigmentation in the BRSMG 715A cultivar.

#### 2.3.4. Genes Involved in Lignin Biosynthesis

Regarding lignin biosynthesis, the *HCT* gene was selected. It encodes shikimate O-hydroxycinnamoyltransferase, one of the main enzymes responsible for lignin biosynthesis, catalyzing two reactions that transform 4-coumaroyl-CoA into 4-coumaroyl-shikimate and caffeoyl shikimate into caffeoyl-CoA. *HCT* was 4× more expressed in the black seed coat cultivar BRSMG 715A (log2FC = 2). In the relative expression analysis via RT-qPCR, this gene showed a significant difference between BRSMG 715A and BRS 413 RR (Figure 4B) but not with DM6563 IPRO. Once again, the expression pattern observed in the RNA-Seq was maintained (Figure 5A). In a study conducted in the seed coat tissue, *HCT* gene was repressed at different stages of seed development [16]. Several studies have shown that *HCT* silencing through RNAi and knockout results in lower lignin content and reduces growth in the plant *Arabidopsis* [64,65,66,67,68]. Higher levels of lignin in the seed are associated with higher physiological quality, reduced damage from moisture, less damage caused by storage fungi, and less mechanical damage [19,69,70]. Additionally, soybean varieties with black seed coats have better storability due to having smaller pores in their membranes and higher lignin contents [20]. The cultivar BRSMG 715A has a high lignin content, which is considered one of the determining factors for higher seed quality [17].

**Figure 6:**
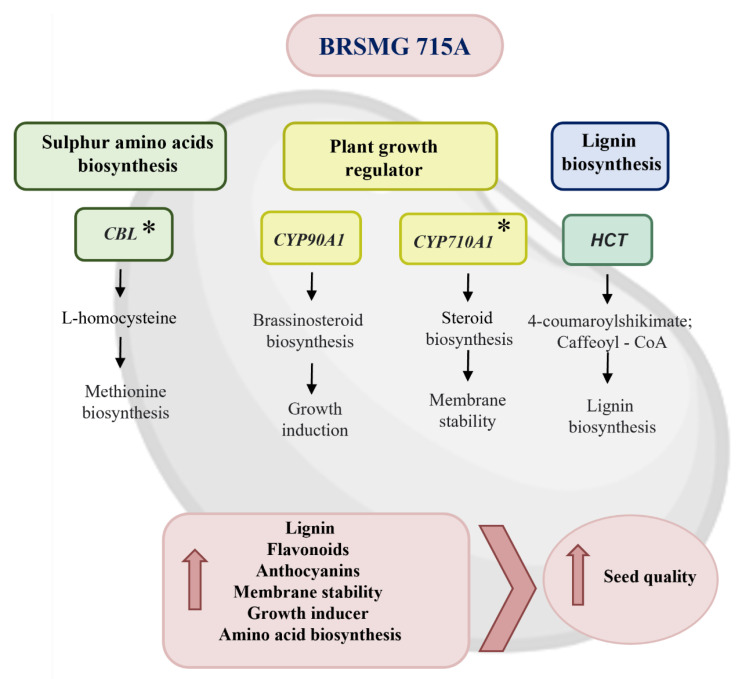
Schematic of the genes significantly induced in the black seed coat cultivar BRSMG 715A of soybeans with black seed coats in relation to the yellow seed coat cultivars BRS 413 RR and DM 6563 IPRO with their respective contributions to increase seed quality and longevity. (*) Asterisks indicate that the gene was induced in comparison to both yellow cultivars.

In summary, the genes induced in the black cultivar are important for seed quality (Figure 6). The *CBL* and *SAHH* enzymes catalyze important reactions for the biosynthesis of sulfur amino acids, especially methionine. Furthermore, the knockout and silencing of the *CBL* gene led to lower embryo development [42,43]. The *SAHH* gene is important for lignin biosynthesis and is necessary for monolignol methylation [40]. *ACS1* and *CYP90A1* are involved in plant hormone biosynthesis and induce germination, while *CYP710A1* is related to plant sterols, which are responsible for membrane stability [47,51,59]. The *ACSF3* gene acts in lipid metabolism and is of great importance for the synthesis of flavonoids and anthocyanin, serving as a substrate for the CHS enzyme [62,63]. The *HCT* gene is responsible for the polymerization of 4-coumarouyl-shikimate and caffeoyl-CoA, and both are necessary for the synthesis of different types of lignin, and gene silencing causes lower lignin content and reduced plant development [66,67,68].

### 2.4. Evaluation of the Number of Copies and Orthologs in Arabidopsis

The genes were also evaluated according to their orthologs, copies found in the soybean genome, and protein domains. The selected genes have two copies, except for *SAHH* (Glyma.08G108800), which has three copies. Further, they present at least 90% similarity with the target gene (Table 2). This similarity between the genes is related to the duplication of the soybean genome, which has approximately 75% of the genes with multiple copies [71]. Duplication is an evolutionary process that can lead to gene functionalization, accumulating copies of non-functional genes (pseudogenes).

Regarding the orthologs found in *Arabidopsis*, only the *CYP710A1* (Glyma.13G217400) and *HCT* (Glyma.02G254600) genes showed direct syntenic orthologs (derived from the same common ancestor through speciation). *CYP710A1* was described in *Arabidopsis* as responsible for the conversion of β-sitosterol into stigmasterol [58,72], and it is likely that the genes have the same function. In *Arabidopsis*, *HCT* has been described to catalyze 4-coumaroyl-CoA into 4-coumaroyl-shikimate and caffeoyl shikimate into caffeoyl-CoA [73,74]. Both genes evaluated present 70% similarity with the ortholog in *Arabidopsis*. The others presented the “Best-hit-arabi-name”, which corresponds to the sequence with the highest physicochemical similarity in *Arabidopsis*, with at least 60% similarity. Further, genes showed conserved protein domains between soybeans and *Arabidopsis*, which means that the genes may display similar functions between these species.

### 2.5. Assessing Lignin Content in the Seed Coat

**Figure 7:**
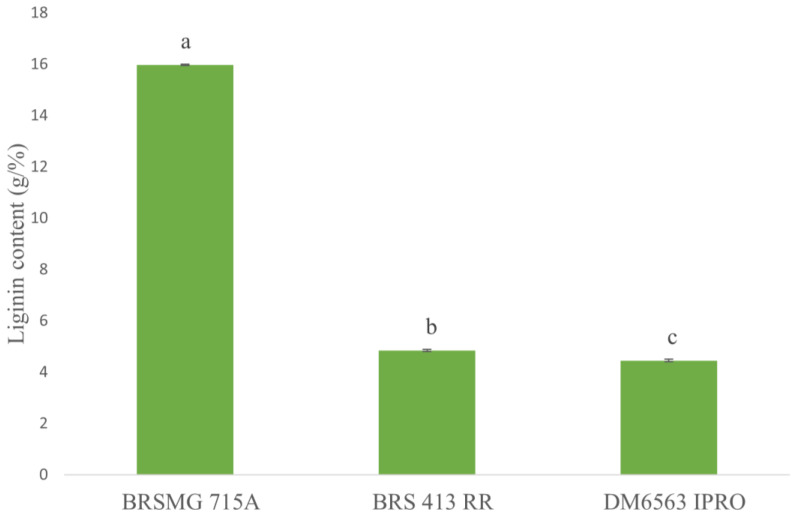
Lignin content in the soybean seed coats of the black seed coat cultivar BRSMG 715A and the yellow-coated cultivars BRS 413 RR and DM 6563 IPRO. Distinct letters show a significant difference via Tukey’s test (*p* ≤ 0.05).

The seeds were evaluated according to the lignin content in their seed coats. The black-coated seed cultivar BRSMG 715A presented the highest lignin content (15.97 g/%) among the cultivars evaluated (Figure 7). Similarly, cultivar BRS 413 RR differed from DM 6563 with a lignin content of 4.84 g/% compared to 4.45 g/% (Figure 7). These results corroborate what was observed in other studies for the BRSMG 715A cultivar and other black seed coat genotypes [9,17,24]. Additionally, cultivars with black seed coats stand out for having a higher concentration of phenolic compounds such as anthocyanin and flavonoids [75]. Furthermore, with high lignin contents, they have lower seed coat permeabilities and absorb less water. Water absorption during storage is directly related to seed deterioration, especially in an uncontrolled environment [18]. Thus, cultivars with black seed coats were associated with better storability and seed longevity, both for their antioxidant potential and lignin content [21,75,76].

## 3. Materials and Methods

For data collection, two identical experiments were carried out in a greenhouse on different dates. The first was used to collect the biological material for RNA-Seq analysis, and the second was used to collect the biological material for RT-qPCR analysis; both experiments were conducted in EMBRAPA-Soja, Londrina, in the years of 2019 and 2021, respectively. The soybean cultivars chosen in this experiment were previously characterized according to their lignin contents, phenolic compounds, and physiological qualities [17,18]. The three cultivars were selected according to their responses to lignin content: BRSMG 715A—black seed coat with high lignin content, BRS 413 RR—yellow seed coat with intermediate lignin content, and DM 6563 IPRO—yellow seed coat with the lowest lignin content; the seeds were provided by EMBRAPA-Soja.

The seeds were germinated on germination paper in alternating periods of light and dark in a germination chamber for 4 days at 28 ± 1 °C and 100% relative humidity (RH). After germination, seedlings with uniform primary roots were transferred to 8-L pots, two per pot. The experiment totaled 33 pots for each cultivar. Each pot contained a 1:1 substrate mixture (fertilized soil and washed sand). The experiment was carried out in a greenhouse under short-day conditions (10 h light/14 h dark) at 28 ± 2 °C and optimal irrigation conditions.

The seeds were harvested when the cultivars reached full maturation and were manually threshed and subsequently homogenized to compose a working sample of 500 g. For each cultivar, the sample was subdivided into six equal parts, which were used to compose the treatments: (a) freshly harvested seeds—FHS and (b) seeds storage in a cold chamber—SCC for 6 months (10 °C and relative humidity 50%), each consisting of 3 repetitions. Furthermore, samples from whole mature seeds were used for total RNA extraction.

### 3.1. RNA-Seq Analysis

Seed samples collected in both treatments were frozen in liquid nitrogen (N2) and stored at −80 °C until RNA extraction. Samples were macerated in liquid N2, and total RNA was extracted using the Concert Plant RNA reagent kit (Invitrogen, Carlsbad, CA, USA) according to the manufacturer’s specifications. RNA quantification was performed in a NanoDrop spectrophotometer according to the following quality and purity parameters: concentration > 600 ng μL^−1^, 260/280 ratio ranging from 1.8 to 2.0, and 260/230 ratio ≥ 2.0.

The RNA was treated with a DNAse-free turbo kit to remove the remaining genomic DNA (Invitrogen, Carlsbad, CA, USA). RNA integrity was assessed via electrophoresis on a 1% (*w*/*v*) agarose gel stained with ethidium bromide (1 μg mL^−1^) [77]. High-quality total RNA samples were sent for sequencing at the University of Georgia (Georgia Genomics Facility—GGF, USA). Before the samples were sequenced, they were evaluated on an Agilent Bioanalyzer 2100 instrument (Agilent Technologies, Santa Clara, CA, USA). Only samples with an RNA integrity number (RIN) ≥ 7.00 were used to synthesize the mRNA-Seq libraries. Libraries were synthesized using an Ilumina TruSeq™ SBS v5 poly-A kit on an Illumina NextSeq 500 1.9 75 bp paired-end sequencing platform (Illumina, San Diego, CA, USA) with at least 1X genome coverage. Each treatment (BRSMG 715A—FHS, BRSMG 715A—SCC, BRS 413 RR—FHS, BRS 413 RR—SCC, DM 6563 IPRO—FHS, and DM 6563 IPRO—SCC) was sequenced in biological triplicate, totaling 18 RNA-seq libraries.

### 3.2. Bioinformatics Analysis

The quality of the raw fragments (FASTQ file format) was assessed using FastQC v.0.11.5 software before and after removing adapters and poor-quality sequences [78,79]. Fragments were cleaned using Trimmomatic software version 0.36 [80], standardizing cuts every four nucleotides at the ends of sequences that had a quality score below 30 (Phred Quality Score, Q ≥ 30).

Fragments were aligned using HISAT2 v.2.1.0 software using the soybean genome Wm82.a2.v1 as a reference [81,82]. PCR artifacts from Illumina sequencing were then removed using Samtools v.1.5 software [83]. Finally, the transcripts were assembled using StringTie v.1.3.3 software [84], and the relative expression levels were obtained from EdgeR v.3.22.3 software [85] via RStudio v.3.5.1 [86].

For each cultivar, the differentially expressed genes were obtained by making comparisons within each treatment (FHS and SCC) between the black seed coat (BRSMG 715A) and the yellow seed coat cultivars (BRS 413 RR; DM 6563 IPRO). Genes with Log2 Fold Change (Log2FC) values ≤−1 and ≥+1 with a false positive rate (FDR) ≤ 0.05 and positive logCPM were considered differentially expressed [87]. To increase the reliability of the analysis, only the differentially expressed genes between the black and yellow cultivars that were conserved in the evaluated treatments were selected for the study.

Gene annotation was performed using the Phytomine tool [81]. Gene enrichment and gene ontology analyses were performed using ShinyGO software based on information from the Kyoto encyclopedia of genes and genomes (KEGG) pathways database [88].

### 3.3. RT-qPCR Analysis

Total RNA was extracted from soybean seed samples using TRIzol^®^ reagent. Then, the samples were treated with the DNAse I kit (Invitrogen, Carlsbad, CA, USA) to remove the remaining DNA. After that, cDNA was synthesized using the Super Script^®^ III First-Strand Synthesis System (Invitrogen, Carlsbad, CA, USA) according to the manufacturer’s instructions. RT-qPCRs were composed of cDNA, 0.2 μM F and R primers, and 1× Plati-num^®^ SYBR Green^®^ qPCR SuperMix UDG reaction buffer (Invitrogen, Carlsbad, CA, USA).

Relative expression was determined in biological and technical triplicates (*n* = 9). Quantitative PCRs were run in a model 7900HT thermocycler (Applied Biosystems, Waltham, MA, USA). The cycling conditions used were denaturation at 95 °C for 20 s, followed by 40 cycles of 95 °C for 3 s, 60 °C for 26 s, and 1 cycle for the melting curve at 95 °C for 15 s, 60 °C for 1 min, and 95 °C for 15 s. Only primers with efficiency ≥85% were considered from the formula E = [10^^(–1/slope) – 1^] [89]. Calibration of gene expression was performed using *β*-actin primers (Glyma.15G050200—F primer 5′GAGCTATGAATTGCCTGATGG3′ and R primer 5′CGTTTCATGAATTCCAGTAGC3′) and *β*-Elf primers (Glyma.13G114700—F primer 5′TTCTGTCTTCTGCAAGTGGTG3′ and R primer 5′GATCCCTCATCCATACATTTCAG3′) [90].

The expression level was determined from the 2^−ΔΔCt^ formula adapted according to primer efficiency [91]. Statistical analysis was performed using the *t*-test (*p* ≤ 0.05) via SasmAgri software (version 8.1) [92]. Pearson’s correlation between RT-qPCR and log2FC RNA-Seq expression was performed via RStudio v.3.5.1 [86].

Seven genes noted as the most highly expressed in seeds of the soybean cultivar BRSMG 715A with black seed coats were selected (Supplementary Material File S1). The genes were chosen using the absolute expression in transcripts per million (TPM) ≥ 3 for each cultivar (BRSMG 715A—black seed coat; DM 6563 IPRO and BRS 413 RR—yellow seed coat) and treatment (FHS, freshly harvested and SCC, cold storage for 6 months). The CDSs of the target genes were obtained from the Phytozome database, and the specific primers were designed using Primer3Plus software (https://primer3plus.com/cgi-bin/dev/primer3plus.cgi (accessed on 4 October 2021)). Homo and heterodimers were eliminated using Multiple Primer Analyzer Software (https://www.thermofisher.com/br/multiple-primer-analyzer.html (accessed on 4 October 2021)).

### 3.4. Lignin Content of Soybean Seed Coat

The lignin content of the soybean seed coat was determined using the acetyl bromide method) [93], which requires 100 seeds of each cultivar with four repetitions. Initially, the seeds were immersed in water for 12 h. Then, the seed coats were removed and dried in an oven at 105 °C for 24 h. The dry matter obtained was ground and homogenized. After that, 300 mg samples of the ground seed coat were separated and centrifuged with different solutions (sodium and potassium phosphate, Triton X-100, 1.0 M NaCl, deionized water, and acetone) to obtain the cell wall. The samples were then placed in a vacuum dryer and subsequently in an oven at 60 °C. The dry samples were macerated, and the protein-free material was obtained. Subsequently, using the acetyl bromide method, 20 mg of cell-wall-lacking proteins was placed in a centrifuge containing 0.5 mL of 25% acetyl bromide (*v*/*v* in glacial acetic acid) and incubated at 70 °C for 30 min [93].

After complete digestion, the sample was quickly cooled in an ice bath and then mixed with 0.9 mL of 2 M NaOH, 0.1 mL of 5 M HCl-hydroxylamine, and a volume of glacial acetic acid to complete the solution. After centrifugation (1400× *g*, 5 min), the absorbance of the supernatant was measured at 280 nm. The results were expressed in mg of lignin per cell wall g^−1^.

### 3.5. Statistical Analyses

The data obtained from the biochemical analysis were subjected to analysis of variance (ANOVA) using RStudio software (version 3.51). When significant differences were found using the *F*-test (*p* < 0.05), the means were compared with the Tukey test (*p* < 0.05).

## 4. Conclusions

The genes induced in the black seed coat cultivar represent several pathways that can be related to the superior seed quality of this cultivar. The *CBL* and *SAHH* enzymes catalyze important reactions for the biosynthesis of sulfur amino acids. The *SAHH* gene also plays a role in lignin biosynthesis and is necessary for monolignol methylation. *ACS1* and *CYP90A1* are involved in ethylene and brassinosteroid biosynthesis, respectively, while *CYP710A1* is related to plant sterols, which are responsible for membrane stability. The *ACSF3* gene acts in lipid metabolism, serving as a substrate for the *CHS* enzyme, which is required for anthocyanin biosynthesis. The *HCT* gene is required for lignin biosynthesis. The BRSMG 715A has almost 4x times more lignin than the yellow seed coat cultivars. These attributes may be related to the higher seed quality of the BRSMG 715A cultivar, which translates to more longevity, resistance to moisture, and mechanical damage.

In the future, gene silencing can be used to evaluate the knockout effect of these genes on phenotypes and to better understand their importance in seed biology, and these results can be later applied in genetic improvement to increase seed longevity.

## Figures and Tables

**Table 1:** Genes that are exclusively expressed in the black seed coat cultivar BRSMG 715A of soybeans.

Gene ID	PhytoMine Annotation	KEGG
Glyma.06G213600	isoamylase/debranching enzyme	sugar metabolism
Glyma.06G043900	cyclin A (CCNA)	seed development
Glyma.01G016700	inositol-pentakisphosphate 2-kinase/ip5 2-kinase	phytic acid biosynthetic pathway
Glyma.02G064300	ribonuclease t2 [ec:3.1.27.1] (e3.1.27.1)	nodule development
Glyma.02G293700	N/A	uncharacterized protein
Glyma.06G267400	leucine-rich repeat-containing protein	disease resistance
Glyma.09G075800	N/A	uncharacterized protein
Glyma.13G194800	leucine-rich repeat-containing protein	disease resistance
Glyma.13G194900	leucine-rich repeat-containing protein	disease resistance
Glyma.16G028300	N/A	uncharacterized protein
Glyma.18G166600	phosphatidylinositol glycan, class s (PIGS)	protein glycosilation
Glyma.20G138000	cysteine-rich repeat secretory protein	disease resistance

**Table 2:** Evaluation of copy number, orthologs, and protein domains between soybeans and *Arabidopsis*.

GeneID	SCN	Copy	%S	Ortholog	%S	Best-Hit-Arabi	S%	Proteic Domain
Glyma.11G228900	2	Glyma.18G028300.1	92	N/A	N/A	AT5G05690.1	80	p450
Glyma.13G217400	2	Glyma.15G095000.1	90	AT2G34500.1	70	N/A	N/A	p450
Glyma.08G204300	2	Glyma.07G019100.1	94	N/A	N/A	AT3G16170.1	61	AMP-binding enzyme
Glyma.03G129700	2	Glyma.19G132000.1	92	N/A	N/A	AT3G57050.1	80	Cys/Met metabolism PLP-dependent enzyme
Glyma.02G254600	2	Glyma.14G061800.1	92	AT2G39980.1	70	N/A	N/A	Transferase family
Glyma.16G032200	2	Glyma.07G065700	96	N/A	N/A	AT3G61510.1	63	Aminotransferase class I and II
Glyma.08G108800	3	Glyma.05G152000.1/Glyma.11G254700.1	99/93	N/A	N/A	AT4G13940.1	93	S-adenosyl-L-homocysteine hydrolase

Legend: SCN—Soybean copy number; S%—Similarity; N/A—Not Available.

## Data Availability

The data will be available in NCBI, BioProject ID: PRJNA950405.

## Supplementary Materials

The following supporting information can be downloaded at: https://www.mdpi.com/article/10.3390/plants12071555/s1. File S1: List of primers used to quantify the relative expression of target genes via RT-qPCR. File S2 showing differently expressed genes in SCC and FHS—Log2FC treatment. File S3: 318 genes differentially expressed in the black cultivar with their respective absolute expressions (TPM). File S4: Attributes of the different genotypes used in the study.
