# Supplementary material for: Transcriptional Profile of Soybean Seeds with Contrasting Seed Coat Color
Source: Plants (Basel). 2023 Apr 4;12(7):1555. doi: 10.3390/plants12071555 (PMC10097363; doi:10.3390/plants12071555)
Supplement: Supplementary file 1 [file plants-12-01555-s001.zip › sup_file1.pdf]

Supplementary Table 1- List of primers used to quantify the relative expression of target genes via RT-qPCR.

Gene ID	Annotation	Primer 5´- 3´	Tm (°C)
Glyma.11G228900	*CYP90A1*	Primer F: CCTGCTCGGAAAACACTCTC<br>Primer R: GAGTTGGCGAAGCTCATTGT	60.0<br>60.4
Glyma.13G217400	*CYP710A*	Primer F: ATGGCTCAATCAGTCCCAAG<br>Primer R: TCTGGGATGTCTGGAGGTTC	60.1<br>60.0
Glyma.08G204300	*ACSF3*	Primer F: ATCTCGGTGGAGCTCGAGTA<br>Primer R: CCACTAAGCCAAATCCCAAG	60.0<br>59.6
Glyma.03G129700	*CBL*	Primer F: AGTTGTGTGCTGAGGGGTTC<br>Primer R: GGAAGCATCATCCACCAAAG	60.2<br>60.5
Glyma.02G254600	*HCT*	Primer F: TTCCCAGACCAAAAGTCCAC<br>Primer R: AAGAGGCATCCCTTTTGGAT	59.9<br>59.9
Glyma.16G032200	*ACS1*	Primer F: ATGAGTGGAGGGGCAACA<br>Primer R: GGGTGGGAATCATAAAAGCA	60.0<br>60.6
Glyma.08G108800	*SAHH*	Primer F: CTATGAGAAGACCGGCGAAC<br>Primer R: GAGACGCTCCTTCATCTTGC	59.8<br>60.1

Legend: CYP90A1: cytochrome P450, family 90, subfamily A, polypeptide 1; **CYP710A**: K09832 - cytochrome P450, family 710, subfamily A; **ACSF3:** malonyl-CoA/methylmalonyl-CoA synthetase, EC.6.2.1.3; **CBL:** cystathionine beta-lyase, (EC.4.4.1.8) -; **HCT**: Shikimate O-hydroxycinnamoyltransferase (EC.2.3.1.133); **ACS1:** 1-aminocyclopropane-1-carboxylate synthase (EC.4.4.1.14); **SAHH:** S-adenosylhomocysteinase hydrolase (EC.3.3.1.1).
